# Application of MALDI-TOF MS to assess clinical characteristics, risk factors, and outcomes associated with anaerobic bloodstream infection: a retrospective observational study

**DOI:** 10.1186/s12941-021-00449-4

**Published:** 2021-06-09

**Authors:** Tsuyoshi Watanabe, Yuki Hara, Yusuke Yoshimi, Waka Yokoyama-kokuryo, Yoshiro Fujita, Masamichi Yokoe, Yoshinori Noguchi

**Affiliations:** 1 Division of Rheumatology, Chubu Rosai Hospital, 2-10-15, Komei-cho, Minato-ku, Nagoya, Aichi 455-8530 Japan; 2 Division of Clinical Laboratory, Japanese Red Cross Nagoya Daini Hospital, Nagoya, Aichi 466-8650 Japan; 3 Division of General Internal Medicine, Japanese Red Cross Nagoya Daini Hospital, Nagoya, Aichi 466-8650 Japan; 4 Division of Nephrology, Chubu Rosai Hospital, 2-10-15, Komei-cho, Minato-ku, Nagoya, Aichi 455-8530 Japan

**Keywords:** Anaerobic blood stream infection (BSI), Matrix-assisted laser desorption ionization–time of flight mass spectrometry (MALDI-TOF MS), *Clostridium* species (spp.)

## Abstract

**Background:**

Correctly identifying anaerobic bloodstream infections (BSIs) is difficult. However, a new technique, matrix-assisted laser desorption ionization-time of flight mass spectrometry (MALDI-TOF MS), enables more accurate identification and appropriate treatment. Anaerobic BSIs identified by MALDI-TOF MS were retrospectively analyzed to determine the clinical and microbiological features and patient outcomes based on the anaerobic genera or group.

**Methods:**

Medical records of patients with anaerobic BSIs were used to conduct a single-center retrospective cohort study from January 2016 to December 2020 in Nagoya, Japan. Multivariate logistic regression analysis was performed to determine the independent risk factors for in-hospital mortality.

**Results:**

Of the 215 patients with anaerobic BSIs, 31 had multiple anaerobic organisms in the blood culture, including 264 total episodes of anaerobic BSIs. *Bacteroides* spp. were isolated the most (n = 74), followed by gram-positive non-spore-forming bacilli (n = 57), *Clostridium* spp. (n = 52), gram-positive anaerobic cocci (GPAC) (n = 27), and gram-negative cocci (n = 7). The median patient age was 76 years; 56.7% were male. The most common focal infection site was intra-abdominal (36.7%). The in-hospital mortality caused by anaerobic BSIs was 21.3%, and was highest with *Clostridium* spp. (36.5%) and lowest with GPAC (3.7%). Age, solid tumors, and *Clostridium* spp. were independent risk factors for in-hospital mortality.

**Conclusions:**

We identified current anaerobic BSI trends using MALDI-TOF MS and reported that mortality in patients with anaerobic BSIs patients was highest with *Clostridium* spp. infections.

## Background

Anaerobic microorganisms are major components of the mucosal membrane microflora in humans, with several hundred species identified by classical molecular methods. Anaerobic bloodstream infections (BSIs) are implicated in 4–10.4% of bacteremic episodes with a high mortality rate (14–25%), even higher in patients who are inappropriately treated [1–4]. From 2000 to 2008, the overall incidence rate of anaerobic BSIs was 8.7 per 100,000 annually [3].

Correct identification of anaerobic BSIs is hampered by their slow growth and numerous taxonomic changes. However, a new technique, matrix-assisted laser desorption ionization-time of flight mass spectrometry (MALDI-TOF MS), correctly identified 91.2% of anaerobic isolates at the species level compared to identification by 16S ribosomal RNA (rRNA) sequencing, which is considered the gold standard [5]. As the mortality rate is higher for patients with inadequate antimicrobial therapy [6], MALDI-TOF MS enables a more accurate microbiologic identification of anaerobic BSIs, allowing for appropriate treatment.

Anaerobic organisms are classified as gram-negative bacilli, gram-negative cocci, gram-positive spore-forming bacilli, gram-positive non-spore-forming bacilli, and gram-positive anaerobic cocci (GPAC) [2, 7]. The majority of anaerobic gram-negative bacilli are *Bacteroides* spp., with *B. fragilis* isolated most often from clinical infections [8–10]. *Clostridium* spp. is the main gram-positive spore-forming bacilli, with *C. perfringens* as the most frequently identified [11, 12]. However, despite epidemiologic and microbiological studies of anaerobic bacteremia using MALDI-TOF MS and 16S rRNA, the clinical characteristics and infection severity of anaerobic genera remain unclear [4, 10, 13].

In this study, we retrospectively analyzed anaerobic BSIs identified by MALDI-TOF MS to determine the clinical and microbiological features and patient outcomes based on the anaerobic genera or group. Furthermore, we conducted a multivariate analysis to evaluate the independent risk factors for in-hospital mortality of anaerobic BSIs. The information presented in this study may be helpful to physicians when diagnosing patients with anaerobic BSIs, and for determining appropriate treatment strategies to improve outcomes in patients with anaerobic BSIs.

## Methods

### Patients and study design

This was a single-center retrospective cohort study conducted from January 2016 to December 2020 at the Japanese Red Cross Nagoya Daini Hospital, an 812-bed tertiary medical center in Nagoya, Japan. This research was approved by the Clinical Research Ethics Committee of the Japanese Red Cross Nagoya Daini Hospital (reference no.1483). As this study does not contain any personal information that could lead to the identification of the patient, the requirement for informed consent was waived by the ethics committee. All subjects were over 18 years of age, and patients diagnosed with anaerobic BSI were eligible for inclusion.

### Data collection

Patients’ data were collected by reviewing electronic medical records. The primary outcome was in-hospital mortality, defined as all-cause mortality at the hospital after index positive blood cultures.

Demographic data included age, sex, body mass index, and community-onset infection (yes or no). Clinical data included fever, steroid use, underlying diseases, indwelling catheter, and comorbidity severities, recorded according to the Charlson Comorbidity Index (CCI) [14]. Laboratory data were also collected, including the white blood cell count (per μL) and creatinine (mg/dL) and lactate (mmol/L) levels. The following information were also collected: the Pitt bacteremia score (PBS) for illness severity in the first 24 h following BSI onset [15], the hospitalization ward, treatments after admission (e.g., vasopressor use, mechanical ventilation, surgical procedures, and appropriate antibiotic prescription), and outcomes (e.g., length of hospital stay and in-hospital mortality). Microbiological data included the likely BSI source, mono- and poly-microorganisms, and the number of bacteremic pathogens (aerobic and anaerobic).

### Species identification and antibiotic sensitivity test

Blood culture samples were incubated for 7 days in the BACT/ALERT 3D instrument (bioMérieux, Marcy-l’Étoile, France) per the manufacturer's protocol. BACT/ALERT FA and FN plus (aerobic and anaerobic) bottles were used for the blood cultures. Subcultures were streaked onto the BBL™ CDC anaerobic 5% sheep blood agar (BD Biosciences, Franklin Lakes, NJ, USA) from the Centers for Disease Control and Prevention and incubated under anaerobic conditions for 2 days. The incubation period was extended if the bacterial growth was insufficient.

Anaerobic bacteria were identified using the MALDI Biotyper (Bruker Daltonics, Inc., Billerica, MA, USA). Bacterial isolates of single colonies were identified using the plate extraction method as previously described by Theel et al. [16]. MALDI-TOF target plates were inoculated with samples from freshly grown bacterial colonies, overlaid with 1 µL of 70% formic acid (FUJIFILM, Tokyo, Japan), and then overlaid with 1 µL matrix (α-cyano-4-hydroxycinnamic acid). The bacterial test standard (Bruker Daltonics, Inc.) was used for instrument calibration. Mass spectra were analyzed in an m/z range of 2000–20,000. MALDI Biotyper software version 3.0 and the most recent MALDI Biotyper libraries were used for bacterial identification.

For each anaerobic bacterial strain, two preparations of colony/sample material were analyzed. The Biotyper software compared each sample's mass spectrum to the reference mass spectrum in the database and calculated an arbitrary unit score value between 0 and 3, reflecting similarities between the sample and reference spectrum, then displayed the top ten matching database records. Log scores were interpreted according to the manufacturer's instructions. Log scores of > 2.0 indicated species identification with high confidence, 1.7–2 indicated low confidence, and < 1.7 was unreliable identification.

### Definitions

Anaerobic BSI was defined by the isolation of obligate anaerobes from one or more sets of aseptically obtained blood culture bottles. Anaerobic polymicrobial BSI was defined as the simultaneous isolation of anaerobic organisms and one or more other organisms from the blood cultures. BSI was considered clinically relevant when a patient had one or more positive anaerobe blood cultures with clinical evidence consistent with infection (e.g., temperature > 38 °C, elevated serum C-reactive protein concentrations [> 40 mg/L], or the infection source was definitively identified). Community-onset bacteremia was defined as an infection acquired outside of the hospital setting with a positive blood culture obtained within 48 h of admission. Nosocomial onset was defined as an infection acquired while being administered treatment within a healthcare setting, or with a positive blood culture obtained more than 48 h after admission with no evidence of infection at admission. Neutropenia was defined as absolute neutrophil counts of 1000/mm^3^ or below when bacteremia occurred. An infection focus was defined by the fulfillment of at least two of the following criteria: bacterial isolation from a focal culture, radiologic or clinical signs of focal infection, or focal infection symptoms. If the BSI source could not be specifically attributed, it was classified as a primary BSI. Initial inappropriate therapy was defined as the administration of empiric antimicrobials that were inactive against the isolated microorganisms, including anaerobes and other causative pathogens, or when an appropriate antimicrobial agent was not administered within 24 h of BSI onset, delaying the administration of antimicrobials. Antibiotic susceptibilities were interpreted according to the Clinical and Laboratory Standards Institute M100 S28 (2018) breakpoints for anaerobes.

### Statistical analysis

SPSS Statistics (version 22; IBM Japan, Tokyo, Japan) was used for statistical analyses. Categorial data and continuous data were expressed as total numbers (percentages) and medians with interquartile ranges (IQRs), respectively. To analyze categorical variables, we used the chi-squared test or Fisher's exact test when appropriate. For continuous variables, we used the Mann–Whitney U test. Logistic regression was used to identify independent risk factors for in-hospital mortality. Known risk factors and factors that were deemed important were included, with or without a low *p*-value. The age, sex, solid tumor, intensive care unit (ICU) stay, *Clostridium* spp., *Bacteroides* spp., and GPAC variables had complete datasets, but the initial inappropriate antibiotics dataset was only 97% complete (n = 208).

## Results

### Patients and isolates

There were 215 patients with anaerobic BSI. Multiple anaerobic organisms were isolated in the blood cultures of 31 out of 215 patients. Therefore, there were 264 total anaerobic BSI episodes (Table 1). In all cases, BSI-causing microorganisms were identified using MALDI-TOF MS. The most frequently isolated anaerobes were *Bacteroides* spp. (n = 74), followed by gram-positive non-spore-forming bacilli (n = 57), *Clostridium* spp. (n = 52), GPAC (n = 27), and gram-negative cocci (n = 7). *B. fragilis* (n = 35) was the most common *Bacteroides* spp. species, as was *Eggerthella lenta* (n = 19) for gram-positive non-spore-forming bacilli, *C. perfringens* (n = 29) for *Clostridium* spp., and *Parvimonas micra* (*P. micra*) (n = 23) for GPAC. Nearly all anaerobic isolates (90%, 237 out of 264) were identified at the species level; 27 isolates were undetermined (13 anaerobic gram-positive bacilli, 5 anaerobic gram-negative bacilli, 4 *Fusobacterium* spp., 2 GPAC, 2 *Clostridium* spp., and 1 *Bacteroides* spp.).

**Table 1:** Anaerobic microorganisms isolated from bloodstream infections

Gram negative			Gram positive		
Bacilli			Spore forming bacilli		
*Bacteroides*		74	*Clostridium*		52
	*fragilis*	35		*perfringens*	29
	*thetaiotaomicron*	15		*ramosum*	5
	*vulgatus*	5		*innocuum*	5
	*uniformis*	5		*clostridioforme*	3
	*ovatus*	4		*paraputrificum*	2
	*cellulosilyticus*	3		*bifermentans*	1
	*caccae*	2		*butyricum*	1
	*stercoris*	2		*cadaveris*	1
	*plebeius*	1		*hathewayi*	1
	*pyogenes*	1		*septicum*	1
	spp	1		*symbiosum*	1
*Fusobacterium*		14		spp	2
	*necrophorum*	6			
	*nucleatum*	2	Non-spore forming bacillis		
	*mortiferum*	2	*Eggerthella lenta*		19
	spp	4	*Lactobacillus*		16
*Parabacteroides*		5		*paracasei*	4
	*distasonis*	4		*fermentum*	2
	*merdae*	1		*gasseri*	2
*Provotella*		2		*rhamnosus*	2
	*buccae*	1		*salivarius*	2
	*melaninogenica*	1	*Actinomyces*		9
				*neuii*	3
Cocci				*odontolyticus*	2
*Veillonella*		7		*oris*	2
	*atypica*	1		*meyeri*	1
	*parvula*	6		*turicensis*	1
			*Bifidobacterium*		6
				*longum*	3
Undermined organism				*breve*	2
Anaerobic gram-negative bacili		5		*adolescentis*	1
			*Cutibacterium*		6
				*acnes*	5
				*avidum*	1
			*Eubacterium limosum*		2
			*Solobacterium moorei*		2
			*Actinotignum schaalii*		1
			*Atopobium parvulum*		1
			*Coprobacillus cateniformis*		1
			Cocci		
			*Parvimonas micra*		23
			*Ruminococcus gnavus*		3
			*Peptoniphilus indolicus*		1
			Undermined organism		
			Anaerobic gram-positive bacili		13
			Anaerobic gram-positive cocci		2
Total		107	Total		157

### Patient and infection characteristics

The median age of anaerobic BSI patients was 76 years (n = 215; interquartile range [IQR], 66–84), 56.7% were male, and community acquisition of anaerobic BSI was 80.9% (Table 2). The most common comorbidity was a solid tumor (28.8%), followed by diabetes mellitus (23.7%), and the median CCI was 5 (IQR, 3–7). Most patients had a fever (68.3%), and the median lactate level was 2.3 mg/dL (IQR, 1.4–3.9). The most common focal infections were intra-abdominal (36.7%), hepatobiliary (12.1%), and oropharyngeal (6.5%); 22.8% had unknown foci of infection. There were 92 patients (42.8%) with polymicrobial BSIs (anaerobic microorganisms with other species).

**Table 2:** Baseline characteristics of patients with anaerobic bloodstream infections

Factor	All cases	*Bacteroides* spp	Non-spore forming bacillis	*Clostridium* spp	GPAC
	(*n* = 215)	(*n* = 60)	(*n* = 57)	(*n* = 52)	(*n* = 27)
Demographics					
Age, median (IQR)	76 (66–84)	74.9 (67.8–84.3)	74 (58–85)	77 (69–83)	83 (69–86)
Male sex	122/215 (56.7)	33/60 (55)	31/57 (54.4)	30/52 (57.7)	14/27 (51.9)
BMI median (IQR)	20.2 (17.8–23.0)	20.4 (18.1–22.2)	19.1 (17.3–23.0)	20.5 (17.7–23.2)	21.1 (18.0–23.3)
Community onset	174/215 (80.9)	47/60 (78.3)	48/57 (84.2)	38/52 (73.1)	27/32 (84.3)
Nosocomial onset	41/215 (19.1)	13/60 (21.7)	9/57 (15.8)	14/52 (26.9)	5/32 (15.6)
Comorbidities					
HIV infection	0	0	0	0	0
ESRD on hemodialysis	13/215 (6.0)	5/60 (8.3)	2/57 (3.5)	3/52 (5.8)	1/27 (3.7)
Diabetes mellitus	51/215 (23.7)	13/60 (21.7)	10/57 (17.5)	18/52 (34.6)	6/27 (22.2)
Cirrhosis	4/215 (1.9)	1/60 (1.7)	1/57 (1.8)	2/52 (3.8)	1/27 (3.7)
CHF	31/215 (14)	5/60 (8.3)	9/57 (15.8)	10/52 (19.2)	3/27 (11.1)
COPD or severe asthma	3/215 (1.4)	0	2/57 (3.5)	1/52 (1.9)	0
Solid tumor	62/215 (28.8)	22/60 (36.7)	14/57 (24.6)	13/52 (25)	8/27 (29.6)
Hematological malignancy	11/215 (5.1)	3/60 (5)	2/57 (3.5)	2/52 (3.8)	1/27 (3.7)
Alcoholism	4/215 (1.9)	0	1/57 (1.8)	1/52 (1.9)	2/27 (7.4)
Steroid use	15/215 (7.0)	5/60 (8.3)	6/57 (10.5)	3/52 (5.8)	1/27 (3.7)
Charlson Comordity Index, median (IQR)	5 (3–7)	5 (4–7)	5 (2–6)	5 (4–7)	5 (3–7)
Characteristics at presentation					
Fever	147/215 (68.3)	38/60 (63.3)	32/57 (56.1)	39/52 (75)	22/27 (81.5)
Neutropenia (ANC < 1000/mL)	9/215 (4.2)	3/60 (5)	1/57 (1.8)	3/52 (5.8)	1/27 (3.7)
Creatinine (mg/dL), median (IQR)	1.0 (0.7–1.5)	1.05 (0.74–1.62)	0.9 (0.6–1.4)	1.2 (0.8–1.8)	0.8 (0.7–1.0)
Lactate (mmol/L), median (IQR)	2.3 (1.4–3.9)	2.4 (1.4–4.2)	2.1 (1.2–3.7)	2.6 (1.8–4.6)	2.2 (1.7–4.4)
Central line catheter	11/215 (5.1)	3/60 (5)	5/57 (8.8)	3/52 (5.8)	0
Urinary catheter	3/215 (1.4)	2/60 (3.3)	0	1/52 (1.9)	0
Primary source					
Oropharyngeal	14/215 (6.5)	0	4/57 (7.0)	0	8/27 (29.6)
Pneumonia	8/215 (3.7)	0	3/57 (5.3)	1/52 (1.9)	1/27 (3.7)
Soft tissue infection	12/215 (5.6)	3/60 (5)	6/57 (10.5)	3/52 (5.8)	0
Central venous catheter	3/215 (1.4)	0	3/57 (5.3)	0	0
Hepatobiliary infection	26/215 (12.1)	5/60 (8.3)	4/57 (7.0)	13/52 (25)	0
Intra-abdominal	79/215 (36.7)	33/60 (55)	20/57 (35.1)	19/52 (36.5)	8/27 (29.6)
Bone and joint	3/215 (1.4)	2/60 (3.3)	0	0	1/27 (3.7)
Endocarditis	1/215 (0.5)	0	0	0	0
Urinary tract infection	9/215 (4.2)	2/60 (3.3)	2/57 (3.5)	1/52 (1.9)	0
Others	11/215 (5.1)	2/60 (3.3)	1/57 (1.8)	4/52 (7.7)	2/27 (7.4)
Primary BSI	49/215 (22.8)	13/60 (21.7)	14/57 (24.6)	11/52 (21.2)	7/27 (25.9)
Bacteremia					
Polymicrobial bacteremia	92/215 (42.8)	29/60 (48.3)	26/57 (45.6)	27/52 (51.9)	15/27 (55.6)
No. of bacteremic pathogen, median (IQR)	1 (1–2)	1 (1–2)	1 (1–2)	1 (1–2)	1 (1–2)
Treatment					
Portion of initial inappropriate treatment	36/208 (17.3)	17/59 (28.8)	15/55 (27.2)	5/50 (10)	5/26 (19.2)
Surgical procedure	51/215 (23.7)	18/60 (30)	13/57 (22.8)	15/52 (28.8)	7/27 (25.9)
ICU care	35/215 (16.3)	11/60 (18.3)	10/57 (17.5)	12/52 (23.1)	4/27 (14.8)
Vasopressor support	43/215 (20.0)	15/60 (25)	11/57 (19.3)	15/52 (28.8)	5/27 (18.5)
Ventilatory support	28/215 (13.0)	8/60 (13.3)	8/57 (14.0)	11/52 (21.2)	5/27 (18.5)
Length of hospital stay, median (IQR)	20 (11–35)	27 (17–40)	18 (10–42)	21 (9–32)	16 (11–33)
Pitt bacteremia score, median (IQR)	1 (0–3)	1.5 (0–4)	1 (0–3)	2 (1–4)	1 (0–3)
Mortality	46/215 (21.3)	16/60 (26.7)	11/57 (19.3)	19/52 (36.5)	1/27 (3.7)

Patients with BSIs caused by *Bacteroides* spp*.* and *Clostridium* spp. often had solid tumors and diabetes mellitus with trans-abdominal and hepatobiliary focal infection sites (Table 2). Patients with GPAC commonly had oropharyngeal (29.6%) and intra-abdominal (29.6%) focal infection sites.

### Treatment and outcome

The in-hospital mortality rate of anaerobic BSI patients was 21.3%. Patients with *Clostridium* spp. BSIs had the highest mortality rate (36.5%), whereas those with GPAC BSIs had the lowest (3.7%). The median hospital stay was 20 days (IQR, 11–35), and inappropriate antibiotics were initially administered to 17.3% of the patients.

Table 3 presents the characteristics of surviving (n = 169) and deceased (n = 46) patients and predictors for death by univariable analysis. Age, diabetes mellitus, CCI, lactate, *Clostridium* spp., vasopressor support, and PBS were associated with in-hospital mortality. Fever, oropharyngeal infection, GPAC, surgical procedure, and length of hospital stay were negatively associated with in-hospital mortality.

**Table 3:** Comparison of clinical characteristics, and outcomes between surviovors and non-survivors

Factor	Survivors (%)	Deceased (%)	*p* value
	(n = 169)	(*n* = 46)	
Demographics			
Age, median (IQR)	75 (64–84)	81 (72–86)	**0.001**
Male sex	96 (56.8)	26 (56.5)	
BMI, median (IQR)	19.8 (18.4–22.1)	19.8 (18.5–22.1)	
Community onset	140 (82.8)	34 (73.9)	
Nosocomial onset	29 (17.2)	12 (26.1)	
Comorbidities			
HIV infection	0	0	
ESRD on hemodialysis	9 (5.3)	4 (8.7)	
Diabetes mellitus	34 (20.1)	17 (37.0)	**0.02**
Cirrhosis	2 (1.2)	2 (4.3)	
CHF	22 (13.0)	9 (19.6)	
COPD or severe asthma	3 (1.8)	0	
Solid tumor	44 (26.0)	18 (39.1)	
Hematological malignancy	8 (4.7)	3 (6.5)	
Alcoholism	3 (1.8)	1 (2.2)	
Steroid use	9 (5.3)	6 (13.0)	
Charlson Comordity Index	4 (3–6)	6 (5–9)	** < 0.001**
Characteristics at presentation			
Fever	125 (74.0)	22 (47.8)	** < 0.001**
Neutropenia	5 (3.0)	4 (8.7)	
Creatinine, median (IQR)	1.0 (0.7–1.4)	1.2 (0.8–2.0)	
Lactate, median (IQR)	2.2 (1.4–3.3)	3 (1.5–7.4)	**0.02**
Central line catheter	9 (5.3)	2 (4.3)	
Urinary catheter	3 (1.8)	0	
Primary source			
Oropharyngeal	14 (8.3)	0	**0.04**
Soft tissue infection	9 (5.3)	3 (6.5)	
Central venous catheter	3 (1.8)	0	
Hepatobiliary infection	20 (11.8)	6 (13.0)	
Intra-abdominal	62 (36.7)	17 (37.0)	
Bone and joint	3 (1.8)	0	
Urinary tract infection	8 (4.7)	1 (2.2)	
Bacteremia			
Polymicrobial bacteremia	70 (40.2)	22 (47.8)	
No. of bacteremic pathogen, median (IQR)	1 (1–2)	1 (1–2)	
*Bacteroides* spp	44 (25.3)	16 (34.8)	
Non-spore forming bacillis	46 (26.4)	11 (23.9)	
*Clostridium* spp	33 (19.0)	19 (41.3)	**0.009**
Treatment			
Initial inappropriate therapy	30 (18.5)	10 (21.7)	
Surgical procedure	47 (27.8)	4 (8.7)	**0.04**
Vasopressor support	28 (16.6)	15 (32.6)	**0.02**
Ventilatory support	20 (11.8)	8 (17.4)	
Length of hospital stay, median (IQR)	24 (15–35)	14 (2–30)	**0.001**
Pitt bacteremia score, median (IQR)	1 (0–3)	3 (1–6)	** < 0.001**

Statistically significant associations are indicated in bold

Multivariable analysis showed that age (adjusted odds ratio [aOR], 1.05; 95% CI, 1.01–1.08), solid tumors (aOR, 2.14; 95% CI, 1.00–4.57), and *Clostridium* spp. (aOR, 3.22; 95% CI, 1.38–7.52) were independent risk factors for in-hospital mortality (Table 4), and GPAC BSIs did not predict a better outcome.

**Table 4:** Risk factors independently associated with death in patients with anaerobic BSI

Variables	Adjusted OR	Multivariable	*p* value
		95% CI	
Age	1.05	1.01–1.08	0.006
Solid tumor	2.14	1.00–4.57	0.05
*Clostridium* spp.	3.22	1.38–7.52	0.005

## Discussion

This study determined the most common BSI-causing anaerobes identified by MALDI-TOF MS at a large tertiary center in Japan. Our results indicated that *Bacteroides* spp. isolates were the most common, followed by anaerobic gram-positive non-spore-forming bacilli and *Clostridium* spp., consistent with previous studies [3, 4]. We detected a higher frequency of anaerobic gram-positive non-spore-forming bacilli than reported in a study conducted from 1993–2004 [2], and *E. lenta* was the most common isolate among anaerobic gram-positive non-spore-forming bacilli in the current study. Although microbiological and epidemiologic data for anaerobic gram-positive non-spore-forming bacilli are lacking, other studies using MALDI-TOF MS and 16S rRNA gene sequencing support our result that *E. lenta* is an important anaerobic BSI-causing microorganism [17, 18]. We found 27 GPAC-induced BSI episodes (10.2% of all cases), which is comparable to the results of Lassmann et al. [2], and *P. micra* was the most common isolate, identical to the findings by Badri M et al. [19].

We found that *Clostridium* spp. BSIs were an independent risk factor for mortality, and the mortality rate was 36.5%. This rate is comparable with the results of Ngo et al. [3] and Rechner et al. [11], who reported rates of 31% and 46%, respectively. To identify BSIs caused by *Clostridium* spp., conventional methods based on biochemical testing are reportedly as reliable as MALDI-TOF MS, meaning that they may have similar ability to evaluate of BSIs caused by *Clostridium* spp. [20]. The advantages of the MALDI-TOF MS protocol are rapid identification and reduced reagent usage and labor costs [20, 21]. Rechner et al. [11] also suggested that the high mortality rate for BSIs caused by *Clostridium* spp. reflected poor health conditions in older and immunocompromised patients. However, Cobo et al. [13] recently reported that a BSI caused by *Clostridium* spp. was not an independent risk factor for anaerobic BSI mortality, but the study had a smaller sample size than ours (n = 136). *C. perfringens* is the most common *Clostridium* spp. and is known to produce a variety of toxins, including lecithinase, a lethal toxin causing massive hemolysis. Although progressive myonecrosis (i.e., gas gangrene) is a life-threatening clostridial infection, there have only been three cases of *Clostridium* spp.-induced soft tissue infections. Of the 19 fatal cases of BSIs caused by *Clostridium* spp., 12 were due to hepatobiliary or intra-abdominal infection. Yang et al. [22] reported that nosocomial acquisition, ICU stay, and CCI were associated with a higher mortality rate in patients having a BSI caused by *C. perfringens*. Additionally, it was reported that *Clostridium* spp. was the contaminant pathogen in 24% of cases after clinical isolation in blood cultures, *C. perfringens* was the contaminant in 27.3% of the cases [22, 23]. Thus, the appropriate initial recognition of the causative *Clostridium* species is essential for treatment, allowing for patients with BSIs caused by *Clostridium* spp. to improve.

In contrast to *Clostridium* spp., the mortality rate of GPAC-induced BSIs was low, at 3.1%. However, GPAC-induced BSIs did not show better outcomes in the multivariable analysis. Earlier studies have shown that the 30-day mortality rates from BSIs caused by GPAC and *P. micra*, the most common GPAC isolate, were low, at 11% and 3.8%, respectively [19, 24]. There were only 46 deaths in this study, which may be an insufficient number to demonstrate a positive association between GPAC-induced BSI and outcome. The effects of other GPAC genera, such as *Anaerococus* spp. and *Peptoniphilus* spp. (which were not identified in this study), should also be considered. Previous reports have indicated that in-hospital mortality rates of BSIs caused by *Anaerococus* spp. and *Peptoniphilus* spp. were low (12% and 5.8%, respectively), and those genera should be included to evaluate association with better outcomes of GPAC-induced BSIs [19]. Thus, future studies should clarify if differences among anaerobic genera or groups are associated with anaerobic BSI prognosis.

We also found that patient-related factors, such as age and solid tumors, were independent risk factors for mortality, consistent with previous reports [13, 25, 26]. However, previously reported risk factors for mortality, such as nosocomial infection, ICU admission, and inappropriate initial treatment, were not associated with in-hospital mortality in this study [13, 19, 27]. The small number of deaths or the effects of unmeasured factors associated with mortality may have underestimated certain mortality risk factors in our study.

This study has some limitations. First, since the study was retrospectively designed, some cases may have been missed, and we could only analyze variables already included in the medical records. Second, our study was hospital-based. Therefore, the risk factors for anaerobic BSIs and mortality relative to the general population were not fully investigated. Third, only 46 deaths were recorded, which may not have sufficient power to adjust for all variables associated with anaerobic BSI prognosis. A large-scale, prospective, and multicenter study is required to accurately investigate anaerobic infection epidemiology and mortality risk factors. Finally, we were unable to demonstrate whether the use of MALDI-TOF as opposed to conventional methods changes the treatment strategies and outcomes due to the retrospective study and lack of microbiological data regarding anaerobic pathogens identified by conventional methods.

## Conclusions

This study presents the current trends for anaerobic BSIs identified by MALDI-TOF MS. *Bacteroides* spp. were the most isolated anaerobes, *Clostridium* spp. affected the mortality rate of patients with anaerobic BSIs, and both genera often had trans-abdominal and hepatobiliary focal infection sites. These results may assist physicians and clinical microbiology laboratories in the diagnosis and treatment of anaerobic BSIs. Further studies are required to elucidate specific differences in anaerobic genera.

## Data Availability

The datasets for the current study are available from the corresponding author on reasonable request.

## Abbreviations

aOR: Adjusted odds ratio
*B. fragilis*: *Bacteroides fragilis*
BSI: Bloodstream infection
CCI: Charlson Comorbidity Index
CI: Confidence interval
C. perfringens: Clostridium perfringens
*E. lenta*: *Eggerthella lenta*
GPAC: Gram-positive anaerobic cocci
INQ: Interquartile range
MALDI-TOF MS: Matrix-assisted laser desorption ionization-time of flight mass spectrometry
PBS: Pitt bacteremia score
*P. micra*: *Parvimonas micra*
rRNA: Ribosomal RNA
